# Predictors and Outcomes of Secondary Prevention Medication in Patients with Coronary Artery Disease Undergoing Percutaneous Coronary Intervention

**DOI:** 10.5334/gh.812

**Published:** 2021-12-27

**Authors:** Tianyu Li, Xiaofang Tang, Ying Song, Yi Yao, Xueyan Zhao, Zhan Gao, Yuejin Yang, Runlin Gao, Bo Xu, Jinqing Yuan

**Affiliations:** 1 Department of Cardiology, Fuwai Hospital, National Centre for Cardiovascular Diseases, Chinese Academy of Medical Sciences and Peking Union Medical College, Beijing, CN

**Keywords:** Secondary prevention medication, guideline adherence, coronary artery disease, percutaneous coronary intervention, prognosis

## Abstract

**Background:**

Evidence on factors associated with guideline-directed secondary prevention medication (GDPM) after percutaneous coronary intervention (PCI) and its effect on the prognosis of patients with coronary artery disease (CAD) is lacking in China.

**Aims:**

To ascertain predictors of GDPM in real-world clinical practice and to assess the effect of GDPM on clinical outcomes.

**Design:**

A retrospective cohort study.

**Methods:**

Consecutive patients admitted to Fuwai Hospital between January 2013 and December 2013 were recruited. GDPM comprised aspirin, clopidogrel, statins, β-blockers, and angiotensin-converting enzyme inhibitors/angiotensin Ⅱ receptor blockers. The primary outcome was five-year major adverse cardiovascular event (MACE) (cardiac death, myocardial infarction [MI] and unplanned revascularization). Multivariable logistic regression was used to identify predictors of prescribing GDPM. Multivariable Cox regression was used to examine the relationship between GDPM and clinical outcomes.

**Results:**

10,067 patients were followed up for a median of 5.0 years (interquartile range: 4.3–5.2), 45.1% were prescribed with GDPM. Presenting with ST-segment elevation MI (adjusted OR = 3.252 [2.832–3.736]), prior MI (adjusted OR = 2.174 [1.948–2.425]), more stents implanted (adjusted OR = 1.063 [1.022–1.106]), overweight (adjusted OR = 1.136 [1.038–1.243]), obesity (adjusted OR = 1.274 [1.100–1.476]), diabetes (adjusted OR = 1.225 [1.115–1.344]), and hypertension (adjusted OR = 3.556 [3.196–3.956]) predicted the prescription of GDPM. Advanced age (adjusted OR = 0.556 [0.379–0.816]) was associated with lower prescription rate of GDPM. Patients with GDPM had lower rate of 5-year MACE (adjusted HR = 0.889 [0.808–0.978]) relative to those without GDPM.

**Conclusions:**

Despite the benefit of GDPM in improving the prognosis of CAD patients undergoing PCI, gaps still exist in GDPM prescription in real-world clinical practice. Our study determined target populations for physicians to strive to promote the application of GDPM.

## Introduction

Coronary artery disease (CAD) is the leading cause of mortality worldwide, accounting for approximately 16% and 18% deaths globally and in China [1,2], respectively. Percutaneous coronary intervention (PCI) plays a crucial role in restoring coronary perfusion and relieving ischaemia for patients with established CAD. Despite the growth in the number of PCI in China over the last decade and the development of treatment strategies, procedural techniques, and novel devices, the mortality of CAD has not declined [2]. The experience in the developed countries has shown that the decrease in deaths from CAD is predominantly attributable to evidence-based medical therapy and reductions in risk factors [3,4]. Comprising appropriate medical therapy, lifestyle interventions, risk factor control, as well as psychosocial and vocational supports [5], secondary prevention is highly recommended as a systemic and lifelong management in all CAD patients to slow disease progression and prevent future events [6,7].

Theoretically, prescribing four classes of cardioprotective drugs recommended by current guidelines, including antiplatelet agents, lipid-lowering drugs, β-blockers and angiotensin-converting enzyme inhibitors/angiotensin Ⅱ receptor blockers (ACEIs/ARBs), is the easiest secondary prevention measure for clinicians to implement and monitor. Although prescription of secondary prevention medication has increased over the past two decades, there remain gaps between guideline and practice [8,9,10]. The effectiveness of this drug combination on reducing cardiovascular events is well proved in developed countries but remains unclear in China [11]. We sought to ascertain predictors of guideline-directed secondary prevention medication (GDPM) in real-world clinical practice, and to assess the effect of GDPM on clinical outcomes, providing evidence from China to help improve the quality of care for CAD patients undergoing PCI.

## Methods

### Study design

The study consecutively recruited CAD patients admitted to Fuwai Hospital, National Centre for Cardiovascular Disease, Beijing, China, for PCI in 2013. Patient demographic, clinical, procedural and angiographic data were collected from the medical record, and follow-up data were collected by an independent group of clinical research coordinators through telephone interviews or by outpatient visits at 1, 6, and 12 months and annually thereafter. The present article was a retrospective analysis of this cohort study and specifically focused on predictors and outcomes of secondary prevention medication in patients with stenting. The study complied with the principles of the Declaration of Helsinki and was approved by the Review Board of Fuwai Hospital.

### Settings and participants

Ten thousand seven hundred and twenty-four consecutive CAD patients undergoing PCI in Fuwai Hospital were recruited from January 2013 to December 2013. Patients with missing prescription data, no stent implantation, and documented contraindication for any secondary prevention drug were excluded from the present study. Contraindication for antiplatelet agents was active pathological bleeding such as peptic ulcer or intracranial haemorrhage. Contraindication for statins was active liver diseases. Contraindications for β-blockers included bradycardia, second/third-degree atrioventricular block, hypotension, and asthma. Contraindications for ACEIs/ARBs were serum creatinine >225 μmol/L and serum potassium >5.0 mmol/L. After specifying the study cohort, we grouped patients by prescription pattern. All patients provided written informed consent before intervention.

### Outcomes and variables

GDPM was defined as prescribing a combination of antiplatelet agents, statins, β-blockers, and ACEIs/ARBs. Considering the large proportion of patients with drug-eluting stents in the study, antiplatelet agents referred to dual antiplatelet therapy with aspirin and clopidogrel. The drugs were administered soon after PCI.

The primary endpoint was a composite endpoint defined as major adverse cardiovascular event (MACE) at five years, including cardiac death, myocardial infarction (MI) and unplanned revascularization. Death that could not be attributed to a noncardiac aetiology was considered as cardiac death. MI was defined by the Third Universal Definition of Myocardial Infarction. Unplanned revascularization was defined as repeat PCI or coronary artery bypass graft of any vessel for ischaemic symptoms and events driven. The secondary endpoint was 30-day MACE. Other endpoints were the individual components of MACE. We also analysed each outcome measure at two years to evaluate the mid-term outcomes.

Age was categorised as ≤65, 66–79 and ≥80 years. Body mass index (BMI, kg/m^2^) was categorised into underweight (<18.5), normal weight (18.5–24.9), overweight (25.0–29.9) and obesity (≥30.0). Hypertension was defined as office systolic blood pressure ≥140 mmHg and/or diastolic blood pressure ≥90mmHg, or a previously established diagnosis. Hyperlipidaemia was defined as total cholesterol ≥6.2 mmol/L, or low-density lipoprotein cholesterol ≥4.1 mmol/L, or total triglyceride ≥2.3 mmol/L according to Chinese Guidelines on Prevention and Treatment of Dyslipidaemia in Adults (2007), or a previously established diagnosis. Laboratory data at the last examination before PCI were used for analysis.

### Statistical methods

Categorical variables are shown as numbers (%). Continuous variables are expressed as mean±standard deviations (SD) or median [interquartile range (IQR)] according to the distribution. Statistical differences were assessed by the Student’ t-test or the Mann-Whitney U-test for continuous variables, and by Pearson’s chi-square test or Fisher’s exact test for categorical variables.

We used multivariable logistic regression to derive the odds ratios (ORs) and corresponding 95% confidence intervals (CIs) for the associations of patient characteristics with the use of GDPM. Variables that were likely to be of clinical importance and those associated with GDPM in univariate analysis (p < 0.1) were considered (Table 1), and a forward stepwise method was applied to arrive at the final model. Linearity in the logit assumption was checked by the Box-Tidwell method and multicollinearity was assessed. We applied the area under the receiver-operating-characteristic curve (AUC) and the calibration plot to evaluate discrimination and calibration of the prediction model, respectively.

**Table 1:** Baseline characteristics for all patients and by prescription pattern.

Patient Characteristics	All patients (n = 10,067)	GDPM (n = 4,540)	Non-GDPM (n = 5,527)	p-value

**Demographic characteristics**				
Age, years	58 ± 10	58 ± 10	58 ± 10.	0.075
≤65, n (%)	7,564 (75.1)	3,398 (74.8)	4,166 (75.4)	0.541
66–79, n (%)	2,374 (23.6)	1,097 (24.2)	1,277 (23.1)	0.213
≥80*^†^, n (%)	129 (1.3)	45 (1.0)	84 (1.5)	**0.019**
Female*, n (%)	2,310 (22.9)	1,002 (22.1)	1,308 (23.7)	0.058
**Clinical characteristics**				
Admission presentation*^†^, n (%)				
Stable CAD	4,021 (39.9)	1,747 (38.5)	2,274 (41.1)	**0.007**
NSTE-ACS	4,709 (46.8)	1,956 (43.1)	2,753 (49.8)	**<0.001**
STEMI	1,337 (13.3)	837 (18.4)	500 (9.0)	**<0.001**
BMI*^†^, kg/m^2^	25.9 ± 3.2	26.2 ± 3.1	25.7 ± 3.20	**<0.001**
<18.5, n (%)	85 (0.8)	32 (0.7)	53 (1.0)	0.166
18.5–24.9, n (%)	3,791 (37.7)	1,568 (34.5)	2,223 (40.2)	**<0.001**
25.0–29.9, n (%)	5,175 (51.4)	2,424 (53.4)	2,751 (49.8)	**<0.001**
≥30.0, n (%)	1,016 (10.1)	516 (11.4)	500 (9.0)	**<0.001**
Current smoke, n (%)	5,740 (57.0)	2,619 (57.7)	3,121 (56.5)	0.219
Diabetes*^†^, n (%)	3,009 (29.9)	1,482 (32.6)	1,527 (27.6)	**<0.001**
Insulin use*, n (%)	1,302 (12.9)	636 (14.0)	666 (12.0)	**0.004**
Hypertension*^†^, n (%)	6,475 (64.3)	3,512 (77.4)	2,963 (53.6)	**<0.001**
Hyperlipidaemia*, n (%)	6,748 (67.0)	3,127 (68.9)	3,621 (65.5)	**<0.001**
COPD, n (%)	230 (2.3)	98 (2.2)	132 (2.4)	0.443
PAD, n (%)	263 (2.6)	116 (2.6)	147 (2.7)	0.743
Prior MI*^†^, n (%)	1,894 (18.8)	1,062 (23.4)	832 (15.1)	**<0.001**
Prior stroke*, n (%)	1,065 (10.6)	516 (11.4)	549 (9.9)	**0.020**
Prior PCI*, n (%)	2,394 (23.8)	1,175 (25.9)	1,219 (22.1)	**<0.001**
Prior CABG*, n (%)	399 (4.0)	187 (4.1)	212 (3.8)	0.469
Haemoglobin, g/L	143 ± 15	144 [133, 154]	144 [133, 154]	0.829
Anaemia, n (%)	351 (3.5)	162 (3.6)	189 (3.4)	0.686
Platelet*, 10^9^/L	200 [168, 236]	204 [171, 240]	197 [165, 233]	**<0.001**
Thrombocytopenia, n (%)	70 (0.7)	29 (0.6)	41 (0.7)	0.536
Total cholesterol*, mmol/L	4.06 [3.45, 4.81]	4.07 [3.45, 4.81]	4.05 [3.44, 4.81]	0.552
HDL-C*, mmol/L	1.00 [0.84, 1.18]	0.98 [0.83, 1.16]	1.01 [0.85, 1.20]	**<0.001**
LDL-C*, mmol/L	2.36 [1.86, 3.02]	2.36 [1.88, 3.00]	2.35 [1.84, 3.03]	0.755
Triglyceride*, mmol/L	1.53 [1.14, 2.11]	1.58 [1.19, 2.16]	1.50 [1.10, 2.07]	**<0.001**
Lp(a), mg/L	186.18 [78.51, 412.98]	188.19 [78.49, 418.59]	184.16 [78.39, 407.55]	0.412
Creatinine*, μmol/L	74.0 [65.3, 83.4]	74.4 [65.6, 84.4]	73.7 [65.1, 82.4]	**<0.001**
eGFR, ml/min	94.1 [83.6, 101.7]	93.8 [82.4, 101.8]	94.4 [84.4, 101.6]	**0.036**
<60.0*	398 (4.0)	196 (4.3)	202 (3.7)	0.090
LVEF, %	64 [60, 67]	63 [59, 67]	64 [60, 68]	**<0.001**
<40*, n (%)	118 (1.2)	66 (1.0)	52 (1.4)	**0.017**
**Angiographic characteristics**				
Multivessel disease*, n (%)	7,252 (72.0)	3,376 (74.4)	3,876 (70.1)	**<0.001**
Left main disease*, n (%)	108 (1.1)	39 (0.9)	69 (1.2)	0.059
Number of stents*^†^	2 [1, 2]	2 [1, 2]	2 [1, 2]	**0.001**
Type of stent				
≥1 DES, n (%)	10,030 (99.6)	4,523 (99.6)	5,507 (99.6)	0.917

Values are mean ± standard deviation, number (%) or median [interquartile range]. GDPM, guideline-directed secondary prevention medication; CAD, coronary artery disease; NSTE-ACS, non-ST-segment elevation acute coronary syndrome; STEMI, ST-segment elevation myocardial infarction; BMI, body mass index; COPD, chronic obstructive pulmonary disease; PAD, peripheral artery disease; MI, myocardial infarction; PCI, percutaneous coronary intervention; CABG, coronary artery bypass graft; HDL-C, high-density lipoprotein cholesterol; LDL-C, low-density lipoprotein cholesterol; Lp(a), lipoprotein (a); eGFR, estimated glomerular filtration rate; LVEF, left ventricular ejection fraction; DES, drug-eluting stent. * Candidate variables selected for the multivariable logistic model. ^†^ Risk-adjusting variables selected for multivariable Cox models.

Cumulative incidences of clinical events were calculated using the Kaplan-Meier method, and comparisons were made with the log-rank test. Cox proportional hazards regression analyses were performed to estimate the hazard ratios (HRs) for prescription pattern comparisons and their 95% CIs. Proportional hazards assumption was tested by computing log-minus-log plots. Variables that clinically related to outcomes or changed the effect estimate by at least 10% when added to the Cox regression model were included in the fully adjusted models (Table 1).

To maximise statistical power and minimize bias due to excluding patients with incomplete data, we used multiple imputation by chained equations to deal with missing values for the main analyses. We generated five imputed datasets, each was independently analysed, and the results were combined using Rubin’s rules. All analyses were repeated with a cohort of 9,254 complete cases as sensitivity analyses.

Two-tailed p-values of <0.05 were considered to be statistically significant. All analyses were conducted with SPSS version 22.0 (IBM, Armonk, NY, USA).

## Results

### Study population and baseline characteristics

**Figure 1:**
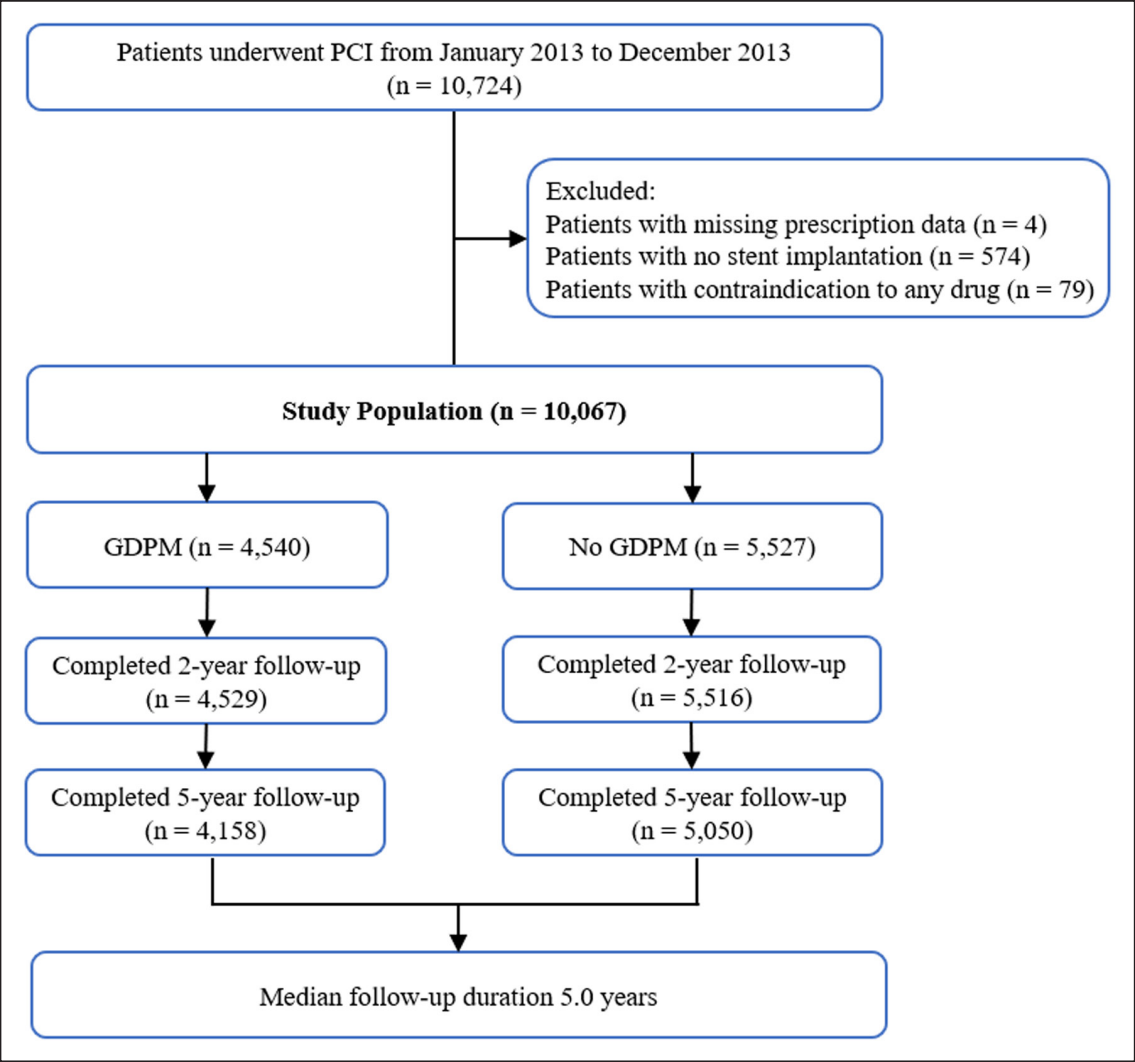
Flow chart of the study population. PCI, percutaneous coronary intervention; GDPM, guideline-directed secondary prevention medication.

Ten thousand seven hundred and twenty–four CAD patients underwent PCI at Fuwai Hospital between January 2013 and December 2013. After excluding patients with missing prescription data (n = 4), no stent implantation (n = 574), and contraindication for any secondary prevention drug (n = 79), the study population comprised 10,067 participants with a median follow-up period of 5.0 years (IQR: 4.3–5.2). Among them, 4,540 (45.1%) patients received GDPM after PCI while 5,527 (54.9%) were not, with 4,529 (99.8%) and 5,516 (99.8%) completed two-year follow-up, respectively; 4,158 (91.6%) and 5,050 (91.4%) completed five-year follow-up, respectively (Figure 1). Patients who were lost to follow-up contributed data to the analyses until the time of loss to follow-up, and their characteristics are shown in Table S1.

Table 1 summarises the characteristics of the participants at baseline. The mean age was 58 ± 10 years, 22.9% were female, 4,021 (39.9%) had a diagnosis of stable coronary artery disease (CAD), 4,709 (46.8%) of non-ST-segment elevation acute coronary syndrome (NSTE-ACS), and 1,337 (13.3%) of ST-segment elevation myocardial infarction (STEMI). Compared with the non-GDPM group, the GDPM group had higher proportions of patients who were overweight and obese, presented with STEMI and multivessel disease, had comorbidities (diabetes, hypertension, or hyperlipidaemia) and a prior history of MI, stroke, or PCI. Patients without GDPM were more likely to be 80 years or older and have lower platelet counts than those in the GDPM group. The proportion of missing data for each variable is small (≤3.2%), and distributions of variables from the pooling of the imputed datasets were similar to those for observed variables (Table S2).

### Medication use and predictors of GDPM

Aspirin was prescribed in 9,940 (98.7%) patients, clopidogrel in 9,920 (98.5%), statins in 9,653 (95.9%), β-blockers in 9,093 (90.3%), and ACEIs/ARBs in 5,268 (52.3%). 9,812 (97.5%) patients had dual antiplatelet therapy with aspirin plus clopidogrel. 4,540 (45.1%) patients received GDPM.

**Figure 2:**
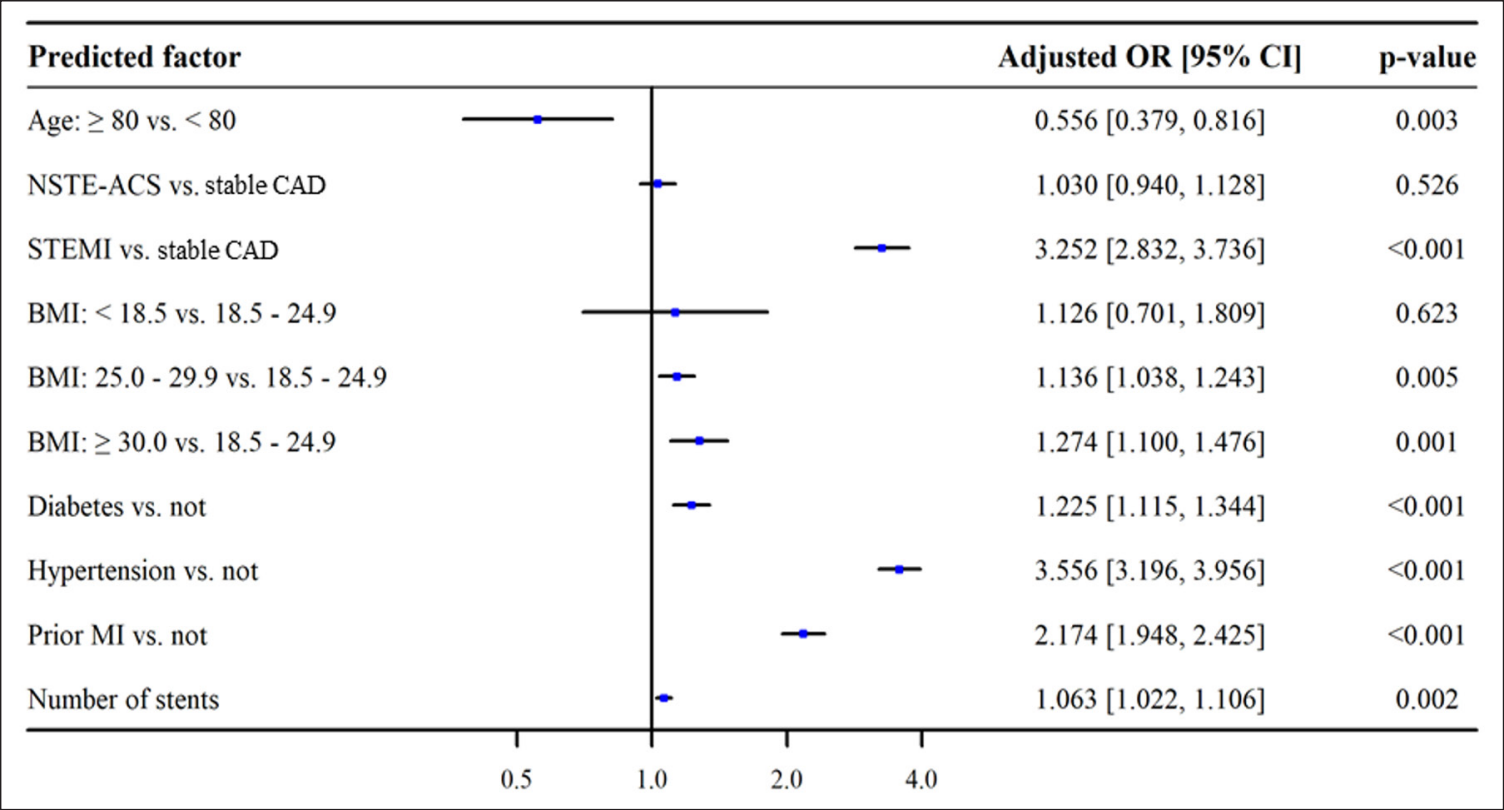
Adjusted ORs and 95% CIs for the likelihood of receiving guideline-directed secondary prevention medication. OR, odds ratio; CI, confidence interval; NSTE-ACS, non-ST-segment elevation acute coronary syndrome; CAD, coronary artery disease; STEMI, ST-segment elevation myocardial infarction; BMI, body mass index; MI, myocardial infarction.

Independent predictors of GDPM are presented in Figure 2. Advanced age (≥80 years) was associated with lower odds of GDPM (adjusted OR = 0.556, 95% CI = 0.379–0.816, p = 0.003). Patients diagnosed as STEMI were more than three times as likely to receive GDPM as stable CAD patients (adjusted OR = 3.252, 95% CI = 2.832–3.736, p < 0.001), and patients who had prior MI were more than twice as likely to receive GDPM as patients without a history of MI (adjusted OR = 2.174, 95% CI = 1.948–2.425, p < 0.001). Higher BMI was associated with an increased likelihood of prescribing GDPM, the adjusted ORs were 1.136 (95% CI = 1.038–1.243, p = 0.005) for overweight and 1.274 (95% CI = 1.100–1.476, p = 0.001) for obesity. Patients with diabetes and hypertension were more likely to receive GDPM, the adjusted ORs were 1.225 (95% CI = 1.115–1.344, p < 0.001) for diabetes and 3.556 (95% CI = 3.196–3.956, p < 0.001) for hypertension. Increased number of stents was associated with higher odds of GDPM (adjusted OR = 1.063, 95% CI = 1.022–1.106, p = 0.002). The prediction model had adequate discrimination with an AUC of 0.683 (95% CI = 0.672–0.693), and satisfactory calibration according to the calibration curve (Figure S1).

### GDPM and clinical outcomes

**Figure 3:**
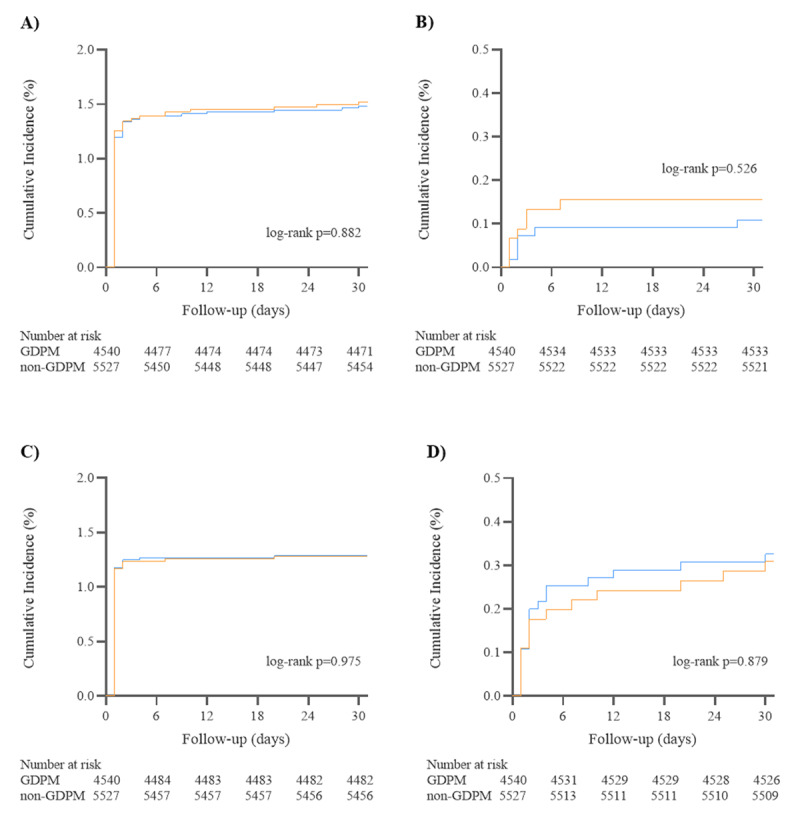
Cumulative incidence curves for 30-day clinical outcomes by prescription pattern **(A–D)** (Orange for GDPM, blue for non-GDPM). Cumulative incidence curves for **A)** MACE, **B)** cardiac death, **C)** MI, **D)** unplanned revascularization. GDPM, guideline-directed secondary prevention medication; MACE, major adverse cardiovascular event; MI, myocardial infarction.

**Figure 4:**
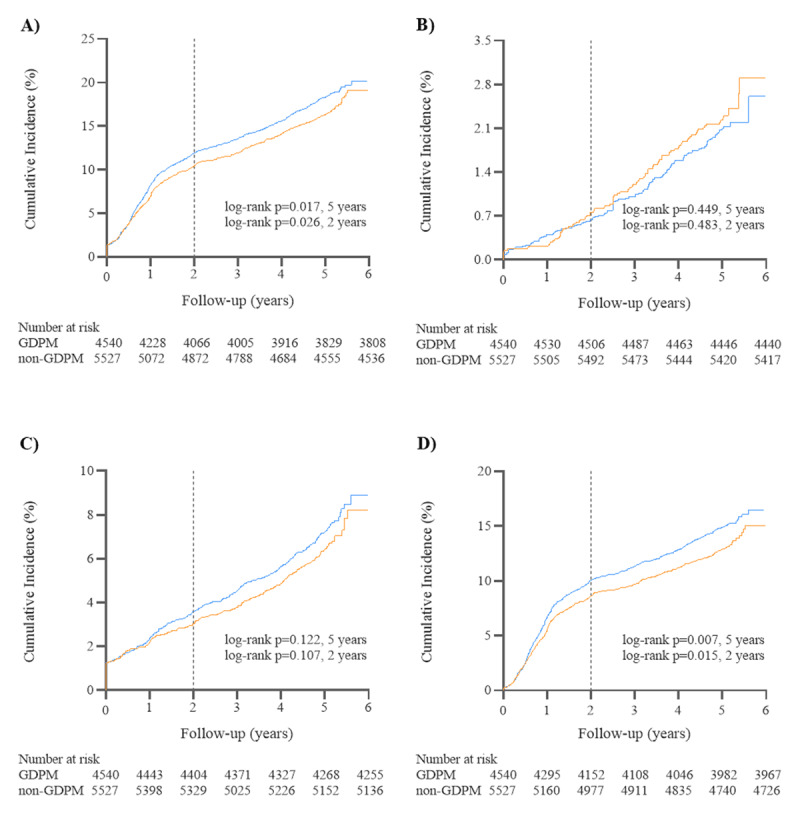
Cumulative incidence curves for five-year clinical outcomes by receipt of GDPM **(A–D)**. (Orange for GDPM, blue for non-GDPM). Cumulative incidence curves for **A)** MACE, **B)** cardiac death, **C)** MI, **D)** unplanned revascularization. GDPM, guideline-directed secondary prevention medication; MACE, major adverse cardiovascular event; MI, myocardial infarction.

Table 2 reports unadjusted and adjusted estimates of the association between GDPM receipt and clinical outcomes. Cumulative incidences of the endpoint events at 5 years and 30 days are described in Figures 3 and 4. At five years, GDPM, as compared with non-GDPM, was associated with significantly reductions in the incidence of MACE (16.1% vs. 17.9%, p = 0.017; unadjusted HR = 0.890, 95% CI = 0.809–0.979, p = 0.017; adjusted HR = 0.889, 95% CI = 0.808–0.978, p = 0.016) and unplanned revascularization (12.6% vs. 14.5%, p = 0.006; unadjusted HR = 0.862, 95% CI = 0.775–0.960, p = 0.007; adjusted HR = 0.864, 95% CI = 0.776–0.962, p = 0.008). The cardiac death rate of the GDPM group didn’t differ significantly from that of the non-GDPM group, nor did MI rate differ significantly between groups.

**Table 2:** Cox regression analysis of GDPM on clinical outcomes.

Outcome measure	GDPM (n = 4,540)	Non-GDPM (n = 5,527)	p-value	Unadjusted HR [95%CI]	p-value	Adjusted HR [95%CI]	p-value

30 days							
MACE	69 (1.5)	82 (1.5)	0.882	1.025 [0.744, 1.411]	0.882	1.019 [0.739, 1.403]	0.910
Cardiac death	7 (0.2)	6 (0.1)	0.526	1.421 [0.478, 4.228]	0.528	1.428 [0.478, 4.266]	0.523
MI	58 (1.3)	71 (1.3)	0.975	0.995 [0.703, 1.407]	0.975	0.991 [0.700, 1.402]	0.959
Unplanned 12revascularization	14 (0.3)	18 (0.3)	0.878	0.947 [0.471, 1.904]	0.879	0.965 [0.479, 1.944]	0.920
2 years							
MACE	474 (10.4)	656 (11.9)	**0.024**	0.875 [0.777, 0.984]	**0.026**	0.875 [0.777, 0.984]	**0.026**
Cardiac death	34 (0.7)	35 (0.6)	0.484	1.184 [0.738, 1.898]	0.483	1.156 [0.721, 1.855]	0.547
MI	136 (3.0)	198 (3.6)	0.102	0.836 [0.672, 1.040]	0.108	0.829 [0.666, 1.032]	0.093
Unplanned 12revascularization	388 (8.5)	551 (10.0)	**0.015**	0.852 [0.748, 0.970]	**0.016**	0.854 [0.750, 0.973]	**0.018**
5 years							
MACE	732 (16.1)	991 (17.9)	**0.017**	0.890 [0.809, 0.979]	**0.017**	0.889 [0.808, 0.978]	**0.016**
Cardiac death	100 (2.2)	110 (2.0)	0.458	1.110 [0.847, 1.455]	0.450	1.089 [0.831, 1.429]	0.536
MI	285 (6.3)	391 (7.1)	0.112	0.887 [0.761, 1.033]	0.123	0.884 [0.759, 1.030]	0.113
Unplanned 12revascularization	573 (12.6)	801 (14.5)	**0.006**	0.862 [0.775, 0.960]	**0.007**	0.864 [0.776, 0.962]	**0.008**

Events are expressed as number (%). GDPM, guideline-directed secondary prevention medication; HR, hazard ratio; CI, confidence interval; MACE, major adverse cardiovascular event; MI, myocardial infarction.

Thirty-day MACE rate in both groups was 1.5%, p = 0.882 (unadjusted HR = 1.025, 95% CI = 0.744–1.411, p = 0.882; adjusted HR = 1.019, 95% CI = 0.739–1.403, p = 0.910). Thirty-day MI rate in both groups was 1.3%, p = 0.975 (unadjusted HR = 0.995, 95% CI = 0.703–1.407, p = 0.975; adjusted HR = 0.991, 95% CI = 0.700–1.402, p = 0.959), accounting for the majority of MACE. Cardiac death and unplanned revascularization were rare and showed no significant difference between groups.

Two-year analyses showed similar results with five-year analyses. MACE occurred in 10.4% patients in the GDPM group versus 11.9% in the non-GDPM group, p = 0.024 (unadjusted HR = 0.875, 95% CI = 0.777–0.984, p = 0.026; adjusted HR = 0.875, 95% CI = 0.777–0.984, p = 0.026). Unplanned revascularization occurred in 8.5% patients in the GDPM group versus 10.0% in the non-GDPM group, p = 0.015 (unadjusted HR = 0.852, 95% CI = 0.748–0.970, p = 0.016; adjusted HR = 0.854, 95% CI = 0.750–0.973, p = 0.018). Cardiac death and MI were not significantly different between the two groups.

### Sensitivity analysis

The baseline characteristics of the complete case cohort were similar to those of the overall study population (Table S3). Prescription rates of individual and combined medications for the complete case cohort are listed in Table S4. The prediction model on GDPM prescription performed well in the internal validation set of complete cases, with an AUC of 0.682 (95% CI = 0.672–0.693), and the calibration curve showed a good calibration (Figure S2). Survival analysis yielded consistent trends with the main analyses (Table S5, Figure S3–S4), however, the difference of two-year outcomes between the two groups didn’t reach statistical significance.

## Discussion

This observational study provided real-world evidence about the predictors and outcomes of GDPM for CAD patients undergoing PCI from China’s biggest cardiovascular centre. More than half of the patients didn’t receive GDPM after PCI. Advanced age was associated with a lower prescription rate of GDPM. Presenting with STEMI, overweight and obesity, history of diabetes or hypertension, prior MI, and implanting more stents were associated with a higher likelihood of GDPM prescription. Patients with GDPM had less MACE at five and two years, but their 30-day clinical outcomes were not improved.

In terms of individual drugs, our study reported higher prescription rates of aspirin, clopidogrel, statins and β-blockers than those reported in other studies [12,13,14]. In contrast, due to the underutilisation of ACEIs/ARBs, the prescription rate of secondary prevention drug combination in our study was relatively low. ACEIs/ARBs were most likely to be omitted, possibly because ACEIs/ARBs have a class Ⅰ indication in patients with hypertension, diabetes, chronic kidney disease, or left ventricular ejection fraction ≤40%, but only a class Ⅱa indication in all other patients, for whom physicians may not routinely prescribed ACEIs/ARBs. Polypills may provide a promising approach to solve the disparity of drug prescription [15].

Consistent with other studies, we found that advanced age was associated with lower prescription rate of GDPM [16,17]. Possibly because of the lack of evidence and perceived elevated risks of using secondary prevention medication in the elderly. A recent study evaluated the effectiveness and safety of secondary prevention medication use in frail older patients and found that whereas the increased risk of functional decline cannot be ruled out, using more drugs can reduce mortality [18]. The survival benefits of GDPM outweigh the risks, especially in those who wish to extend their lives.

Our results support the finding of previous work that increased disease severity and cardiovascular risk factors were associated with higher prescription rate GDPM [12,14]. Patients with STEMI or prior MI were more likely to receive GDPM. Implanting more stents, indicating more advanced atherosclerosis, predicted a higher likelihood of GDPM prescription. Accompanied by cardiovascular risk factors such as overweight and obesity, diabetes, and hypertension also related to GDPM prescription. These results may be explained by the fact that physicians usually pay more attention to patients in more severe disease status or accompanied by more risk factors, and take more aggressive treatment for them because these patients are expected to derive the most clinical benefit through combination evidence-based medical therapy [19]. On the contrary, some studies reported a treatment-risk paradox that high-risk patients tended to receive less secondary prevention medications [20,21]. This inconsistency may be attributed to heterogeneity among studies (e.g., patient population, physicians’ attitudes, clinical settings, health systems, etc.). Targeted improvement measures are needed based on specific settings of individual countries and hospitals.

We didn’t observe the effect of GDPM on reducing short-term events. Approximately 90% of the 30-day events occurred within two days after PCI; among them, 95% were periprocedural MI. Other events were too rare to reach statistical significance. The predominant mechanisms of periprocedural MI are procedural-related mechanical causes such as side-branch occlusion [22], which are hard to be prevented by secondary prevention drugs. However, our study indicated that GDPM provided a better mid- and long-term prognosis. Notably, the reduction of MACE was mainly due to less unplanned revascularization, which means the improvement of patients’ quality of life after PCI and the reduction of healthcare costs. However, we have not proven the findings of other studies that GDPM was associated with reduced mortality or MI [11,23]. A possible explanation might be that we advised all patients to return for coronary angiography at discharge and each follow-up if indicated by symptoms or objective evidence of myocardial ischemia. Patients might receive revascularization when symptoms or ischemic evidence appeared but had not yet developed MI or cardiac death, reducing the likelihood of MI and cardiac death. Alternatively, the increased disease severity and risk factors among patients in the GDPM group might partially offset the benefits of GDPM even after balancing baseline differences between groups.

The study has some limitations. First, as an observational study, the possibility of residual confounding exists due to unknown and unmeasured potential confounders (e.g., education level, income, insurance status), which may relate to prescription and prognosis. Second, the single-centre study conducted in a tertiary teaching cardiovascular hospital. Physicians at our centre may have better understanding and adherence of guidelines than physicians in other hospitals, especially those in rural areas, the actual GDPM prescription rate in the country may be more insufficient. In China, the prevalence of cardiovascular disease has been higher in rural areas than in urban areas for years, which further reflects the vast room for improvement in secondary prevention, and a large proportion of CAD patients can benefit from quality improvement program. Third, although current guidelines recommend ticagrelor and prasugrel, the P2Y_12_ inhibitor used in the study was clopidogrel in accordance with the guidelines at that time, which may limit the generalizability of this study. Forth, we have no data regarding follow-up prescriptions and patients’ adherence, which reduces the accuracy of the interpretation of GDPM’s impact on clinical outcomes. Last, this study only focused on medications without considering other secondary prevention interventions recommended by guidelines, such as physical activity, smoking cessation, healthy diet and cardiac rehabilitation, which requires further research.

## Conclusion

Despite the benefit of GDPM in preventing mid- and long-term MACE for CAD patients undergoing PCI, GDPM remains vastly underutilised in China, mainly driven by the underutilisation of ACEIs/ARBs. Paying more attention to patients with specific characteristics helps to increase the application of GDPM, thereby improving the prognosis of CAD patients undergoing PCI.

## Additional File

The additional file for this article can be found as follows:

10.5334/gh.812.s1 Supplementary materials. Supplementary Figures and Tables.
