# Supplementary material for: Predictors and Outcomes of Secondary Prevention Medication in Patients with Coronary Artery Disease Undergoing Percutaneous Coronary Intervention
Source: Glob Heart. 2021 Dec 27;16(1):89. doi: 10.5334/gh.812 (PMC8719473; doi:10.5334/gh.812)
Supplement: Supplementary materials. — Supplementary Figures and Tables. [file gh-16-1-812-s1.pdf]

Supplementary materials for:

Predictors and Outcomes of Secondary Prevention Medication in Patients with Coronary Artery Disease Undergoing Percutaneous Coronary Intervention

Tianyu Li, MD, Xiaofang Tang, MD, Ying Song, MD, Yi Yao, MD, Xueyan Zhao, MD, Zhan Gao, MD, Yuejin Yang, MD, Runlin Gao, MD, Bo Xu, MBBS, Jinqing Yuan, MD

Affiliation:
Department of Cardiology, Fuwai Hospital, National Centre for Cardiovascular Diseases, Chinese Academy of Medical Sciences and Peking Union Medical College, Beijing 100037, China.

Table S1 Characteristics for patients completed and lost to 2-year and 5-year follow-up.

Patient characteristics	2-year			5-year		
	Completed follow-up (n = 10045)	Lost to follow-up (n = 22)	p-value	Completed follow-up (n = 9208)	Lost to follow-up (n = 859)	p-value
**Demographic characteristics**						
Age, years	58 ± 10	58 ± 10.	0.761	58 ± 11	58 ± 10	0.102
≤65, n (%)	7548 (75.1)	6 (72.7)	0.794	6909 (75.0)	655 (76.3)	0.429
66-79, n (%)	2369 (23.6)	5 (22.7)	0.925	2181 (23.7)	193 (22.5)	0.421
≥80, n (%)	128 (1.3)	1 (4.5)	0.247	118 (1.3)	11 (1.3)	0.998
Female, n (%)	2307 (23.0)	3 (13.6)	0.299	2118 (23.0)	192 (22.4)	0.665
**Clinical characteristics**						
Admission presentation, n (%)						
Stable CAD	4009 (39.9)	12 (54.5)	0.162	3707 (40.3)	314 (36.6)	**0.034**
NSTE-ACS	4702 (46.8)	7 (31.8)	0.156	4300 (46.7)	409 (47.6)	0.607
STEMI	1334 (13.3)	3 (13.6)	1.000	1201 (13.0)	136 (15.8)	**0.021**
BMI, kg/m^2	25.9 ± 3.2	25.9 ± 3.1	0.978	25.9 ± 3.2	25.9 ± 3.5	0.567
<18.5, n (%)	85 (0.8)	0 (0)	1.000	71 (0.8)	14 (1.6)	**0.009**
18.5-24.9, n (%)	3783 (37.7)	8 (36.4)	1.000	3457 (37.5)	334 (38.9)	0.439
25.0-29.9, n (%)	5163 (51.4)	12 (54.5)	0.768	4759 (51.7)	416 (48.4)	0.068
≥30.0, n (%)	1014 (10.1)	2 (9.1)	1.000	921 (10.0)	95 (11.1)	0.325
Current smoke, n (%)	5729 (57.0)	11 (50.0)	0.506	5241 (56.9)	499 (58.1)	0.507
Diabetes, n (%)	3007 (29.9)	2 (9.1)	**0.033**	2756 (29.9)	253 (29.5)	0.770
Insulin use, n (%)	1165 (12.0)	1 (4.5)	0.507	1076 (12.1)	90 (10.9)	0.321
Hypertension, n (%)	6461 (64.3)	14 (63.6)	0.947	5924 (64.3)	551 (64.1)	0.911
Hyperlipidaemia, n (%)	6731 (67.0)	17 (77.3)	0.306	6170 (67.0)	578 (67.3)	0.867
COPD, n (%)	230 (2.3)	0 (0)	1.000	210 (2.3)	20 (2.3)	0.929
PAD, n (%)	263 (2.6)	0 (0)	1.000	237 (2.6)	26 (3.0)	0.426
Prior MI, n (%)	1890 (18.8)	4 (18.2)	1.000	1740 (18.9)	154 (17.9)	0.487
Prior stroke, n (%)	1063 (10.6)	2 (9.1)	1.000	969 (10.5)	96 (11.2)	0.552
Prior PCI, n (%)	2388 (23.8)	6 (27.3)	0.700	2203 (23.9)	191 (22.2)	0.266
Prior CABG, n (%)	398 (4.0)	1 (4.5)	0.590	365 (4.0)	34 (4.0)	0.993
Hemoglobin, g/L	144 [133, 154]	146 [138, 155]	0.425	144 [133, 154]	144 [132, 154]	0.663

Anaemia, n (%)	351 (3.5)	0 (0)	1.000	327 (3.6)	24 (2.8)	0.247
Platelet, 10^9/L	199 [168, 236]	199 [172, 247]	0.899	199 [167, 236]	202 [169, 237]	0.316
Thrombocytopenia, n (%)	70 (0.7)	0 (0)	1.000	64 (0.7)	6 (0.7)	0.991
Total cholesterol, mmol/L	4.05 [3.44, 4.81]	3.91 [3.22, 4.51]	0.182	4.05 [3.45, 4.81]	4.06 [3.38, 4.72]	0.058
HDL-C, mmol/L	0.99 [0.84, 1.17]	0.96 [0.80,1.06]	0.137	1.00 [0.84, 1.17]	0.96 [0.80, 1.16]	**<0.001**
LDL-C, mmol/L	2.35 [1.86, 3.00]	2.14 [1.90, 2.59]	0.153	2.35 [1.86, 3.02]	2.33 [1.84, 2.92]	**0.041**
Triglyceride, mmol/L	1.53 [1.14,2.10]	1.68 [1.27, 2.34]	0.221	1.53 [1.14, 2.09]	1.55 [1.15, 2.18]	0.717
Lp(a), mg/L	183.79 [78.34, 407.78]	211.45 [61.65, 448.25]	0.749	183.17 [78.46, 406.72]	189.94 [77.10, 416.73]	0.801
Creatinine, μmol/L	73.9 [65.2, 83.4]	71.2 [65.5, 81.0]	0.944	74.0 [65.3, 83.4]	72.6 [64.7, 83.6]	0.285
eGFR, ml/min	94.2 [83.6, 101.8]	96.7 [87.0, 102.2]	0.761	94.1 [83.6, 101.7]	95.0 [83.5, 102.3]	0.079
<60.0	397 (4.0)	1 (4.5)	0.589	372 (4.0)	26 (3.0)	0.145
LVEF, %	64 [60, 67]	64 [58, 70]	0.913	64 [60, 67]	64 [60, 68]	0.730
<40, n (%)	116 (1.2)	2 (9.5)	**0.026**	104 (1.2)	14 (1.7)	0.186
**Angiographic characteristics**						
Multivessel disease, n (%)	7237 (72.0)	15 (68.2)	0.687	6647 (72.2)	605 (70.4)	0.273
Left main disease, n (%)	108 (1.1)	0 (0)	1.000	103 (1.1)	5 (0.6)	0.144
Number of stents	2 [1, 2]	1 [1, 2]	0.159	2 [1, 2]	2 [1, 2]	0.437
Type of stent						
≥1 DES, n (%)	10008 (99.6)	22 (100.0)	1.000	9173 (99.6)	857 (99.8)	0.495

Values are mean±standard deviation, number (%) or median [interquartile range].

GDPM, guideline-directed secondary prevention medication; CAD, coronary artery disease; NSTE-ACS, non-ST-segment elevation acute coronary syndrome; STEMI, ST-segment elevation myocardial infarction; BMI, body mass index; COPD, chronic obstructive pulmonary disease; PAD, peripheral artery disease; MI, myocardial infarction; PCI, percutaneous coronary intervention; CABG, coronary artery bypass graft; HDL-C, high-density lipoprotein cholesterol; LDL-C, low-density lipoprotein cholesterol; Lp(a), lipoprotein (a); eGFR, estimated glomerular filtration rate; LVEF, left ventricular ejection fraction; DES, drug-eluting stent.

Table S2 Distribution of variables with missing data comparing observed complete case data to results from the pooling of the datasets with imputed variables from multiple imputation.

Variable	Unit	Number (%) with missing data	Complete case	Multiple imputation
Insulin use	n (%)	325 (3.2)	1166 (12.0)	1302 (12.9)
Total cholesterol, mmol/L	Median [IQR]	256 (2.5)	4.06 [3.45, 4.81]	4.06 [3.45, 4.81]
HDL-C, mmol/L	Median [IQR]	256 (2.5)	0.99 [0.84, 1.18]	1.00 [0.84, 1.18]
LDL-C, mmol/L	Median [IQR]	256 (2.5)	2.35 [1.86, 3.01]	2.36 [1.86, 3.02]
Triglyceride, mmol/L	Median [IQR]	256 (2.5)	1.53 [1.15, 2.10]	1.53 [1.14, 2.11]
Lp(a), mg/L	Median [IQR]	259 (2.6)	184.29 [78.38, 410.18]	186.18 [78.51, 412.98]
LVEF, %	Median [IQR]	257 (2.6)	64 [60, 67]	64 [60, 67]
<40	n (%)	257 (2.6)	118 (1.2)	118 (1.2)

HDL-C, high-density lipoprotein cholesterol; LDL-C, low-density lipoprotein cholesterol; Lp(a), lipoprotein (a); LVEF, left ventricular ejection fraction; IQR, interquartile range.

Table S3 Baseline characteristics for complete cases and by prescription pattern.

Patient characteristics	Complete cases (n = 9,254)	GDPM (n = 4,191)	Non-GDPM (n = 5,063)	p-value
**Demographic characteristics**				
Age, years	58 ± 10	58 ± 10	58 ± 10	0.077
≤65, n (%)	6,958 (75.2)	3,134 (74.8)	3,824 (75.5)	0.406
66-79, n (%)	2,181 (23.6)	1,018 (24.3)	1,163 (23.0)	0.137
≥80, n (%)	115 (1.2)	39 (0.9)	76 (1.5)	**0.014**
Female, n (%)	2,106 (22.8)	916 (21.9)	1,190 (23.5)	0.060
**Clinical characteristics**				
Admission presentation, n (%)				
Stable CAD	3,677 (39.7)	1,603 (38.2)	2,074 (41.0)	**0.008**
NSTE-ACS	4,327 (46.8)	1,796 (42.9)	2,531 (50.0)	**<0.001**
STEMI	1,250 (13.5)	792 (18.9)	458 (9.0)	**<0.001**
BMI, kg/m^2	25.9 ± 3.2	26.1 ± 3.1	25.7 ± 3.2	**<0.001**
<18.5, n (%)	78 (0.8)	30 (0.7)	48 (0.9)	0.224
18.5-24.9, n (%)	3,517 (38.0)	1,468 (35.0)	2,049 (40.5)	**<0.001**
25.0-29.9, n (%)	4,731 (51.1)	2,222 (53.0)	2,509 (49.6)	**0.001**
≥30.0, n (%)	928 (10.0)	471 (11.2)	457 (9.0)	**<0.001**
Current smoke, n (%)	5,288 (57.1)	2,421 (57.8)	2,867 (56.6)	0.270
Diabetes, n (%)	2,567 (27.7)	1,276 (30.4)	1,291 (25.5)	**<0.001**
Insulin use, n (%)	1,111 (12.0)	546 (13.0)	565 (11.2)	**0.006**
Hypertension, n (%)	5,911 (63.9)	3,219 (76.8)	2,692 (53.2)	**<0.001**
Hyperlipidaemia, n (%)	6,197 (67.0)	2,885 (68.8)	3,312 (65.4)	**<0.001**
COPD, n (%)	213 (2.3)	94 (2.2)	119 (2.4)	0.731
PAD, n (%)	242 (2.6)	109 (2.6)	133 (2.6)	0.938
Prior MI, n (%)	1,747 (18.9)	977 (23.3)	770 (15.2)	**<0.001**
Prior stroke, n (%)	961 (10.4)	470 (11.2)	491 (9.7)	**0.017**
Prior PCI, n (%)	2,197 (23.7)	1,079 (25.7)	1,118 (22.1)	**<0.001**
Prior CABG, n (%)	366 (4.0)	174 (4.2)	192 (3.8)	0.377
Hemoglobin, g/L	143 ± 15	143 ± 15	143 ± 16	0.595

Anaemia, n (%)	325 (3.5)	151 (3.6)	174 (3.4)	0.665
Platelet, 10^9/L	200 [168, 236]	204 [171, 240]	197 [166, 270]	**<0.001**
Thrombocytopenia, n (%)	66 (0.7)	28 (0.7)	38 (0.8)	0.639
Total cholesterol, mmol/L	4.06 [3.45, 4.81]	4.06 [3.45, 4.81]	4.05 [3.44, 4.81]	0.602
HDL-C, mmol/L	0.99 [0.84, 1.18]	0.98 [0.83, 1.16]	1.01 [0.85, 1.20]	**<0.001**
LDL-C, mmol/L	2.35 [1.86, 3.01]	2.36 [1.89, 2.99]	2.35 [1.84, 3.02]	0.524
Triglyceride, mmol/L	1.53 [1.14, 2.10]	1.57 [1.18, 2.15]	1.50 [1.10, 2.05]	**<0.001**
Lp(a), mg/L	183.71 [78.67, 407.10]	187.21 [78.89, 415. 60]	179.08 [78.64, 400,46]	0.180
Creatinine, μmol/L	73.91 [65.32, 83.40]	74.37 [65.66, 84.35]	73.54 [65.02, 82.34]	**<0.001**
eGFR, ml/min	94.16 [83.58, 101.77]	93.80 [82.52, 101.88]	94.50 [84.55, 101.67]	**0.033**
<60.0	358 (3.9)	179 (4.3)	179 (3.5)	0.068
LVEF, %	64 [60, 67]	63 [59, 67]	64 [60, 68]	**<0.001**
<40, n (%)	105 (1.1)	58 (1.4)	47 (0.9)	**0.039**
**Angiographic characteristics**				
Multivessel disease, n (%)	6,663 (72.0)	3,106 (74.1)	3,557 (70.3)	**<0.001**
Left main disease, n (%)	95 (1.0)	38 (0.9)	57 (1.1)	0.298
Number of stents	2 [1, 2]	2 [1, 2]	2 [1, 2]	**0.003**
≥1 DES, n (%)	9,220 (99.6)	4,176 (99.6)	5,044 (99.6)	0.891

Values are mean±standard deviation, number (%) or median [interquartile range].

GDPM, guideline-directed secondary prevention medication; CAD, coronary artery disease; NSTE-ACS, non-ST-segment elevation acute coronary syndrome; STEMI, ST-segment elevation myocardial infarction; BMI, body mass index; COPD, chronic obstructive pulmonary disease; PAD, peripheral artery disease; MI, myocardial infarction; PCI, percutaneous coronary intervention; CABG, coronary artery bypass graft; HDL-C, high-density lipoprotein cholesterol; LDL-C, low-density lipoprotein cholesterol; Lp(a), lipoprotein (a); eGFR, estimated glomerular filtration rate; LVEF, left ventricular ejection fraction; DES, drug-eluting stent.

Table S4 Prescription rates of individual and combined medications for complete cases.

Individual and combined medications	Prescription rate, n (%)
Individual medication	
Aspirin	9,142 (98.8)
Clopidogrel	9,119 (98.5)
Statins	8,881 (96.0)
β-blocker	8,372 (90.5)
ACEIs/ARBs	4,854 (52.5)
Combined medications	
DAPT	9,025 (97.5)
GDPM	4,191 (45.3)

ACEI, angiotensin-converting enzyme inhibitor; ARB, angiotensin Ⅱ receptor blocker; DAPT, dual antiplatelet therapy; GDPM, evidenced-based medications.

Table S5 Cox regression analysis of GDPM on clinical outcomes for complete cases.

Outcome measure	GDPM (n = 4178)	Non-GDPM (n = 5076)	p-value	Unadjusted HR [95%CI]	p-value	Adjusted HR [95%CI]	p-value
30 days							
MACE	60 (1.4)	72 (1.4)	0.943	1.012 [0.719, 1.426]	0.943	1.004 [0.713, 1.415]	0.982
Cardiac death	3 (0.1)	4 (0.1)	1.000	0.911 [0.204, 4.071]	0.903	0.821 [0.181, 3.734]	0.799
MI	53 (1.3)	64 (1.3)	0.974	1.006 [0.699, 1.448]	0.974	0.991 [0.700, 1.402]	0.959
Unplanned revascularization	13 (0.3)	17 (0.3)	0.841	0.929 [0.451, 1.912]	0.841	0.999 [0.694, 1.439]	0.997
2 years							
MACE	442 (10.6)	601 (11.8)	0.056	0.890 [0.787, 1.006]	0.062	0.889 [0.786, 1.005]	0.060
Cardiac death	27 (0.6)	32 (0.6)	0.924	1.026 [0.615, 1.712]	0.923	1.001 [0.600, 1.672]	0.995
MI	125 (3.0)	184 (3.6)	0.092	0.825 [0.657, 1.035]	0.097	0.817 [0.651, 1.025]	0.081
Unplanned revascularization	367 (8.8)	506 (10.0)	0.052	0.877 [0.766, 1.003]	0.055	0.878 [0.768, 1.005]	0.058
5 years							
MACE	682 (16.3)	910 (17.9)	**0.042**	0.903 [0.817, 0.997]	**0.043**	0.901 [0.816, 0.995]	**0.040**
Cardiac death	88 (2.1)	100 (2.0)	0.644	1.070 [0.804, 1.425]	0.643	1.048 [0.787, 1.396]	0.747
MI	263 (6.3)	356 (7.0)	0.169	0.896 [0.764, 1.051]	0.177	0.892 [0.760, 1.046]	0.159
Unplanned revascularization	540 (12.9)	736 (14.5)	**0.029**	0.884 [0.791, 0.988]	**0.029**	0.885 [0.792, 0.989]	**0.031**

Cumulative incidences of outcome measures are number (%).

GDPM, guideline-directed secondary prevention medication; HR, hazard ratio; CI, confidence interval; MACE, major adverse cardiovascular event; MI, myocardial infarction.

A)

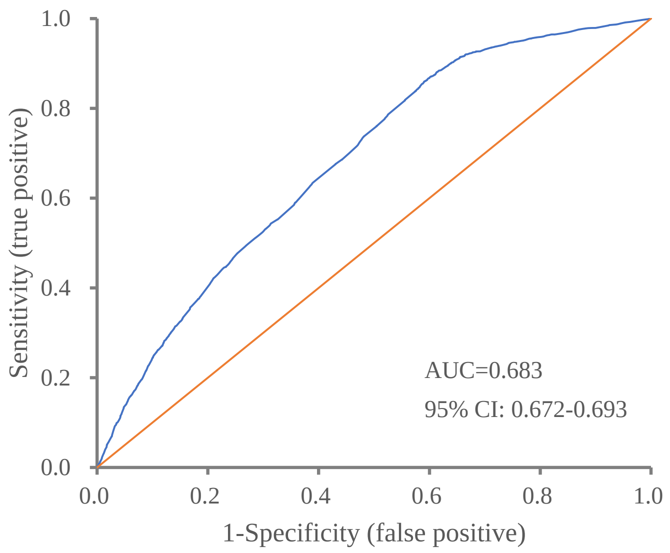

B)

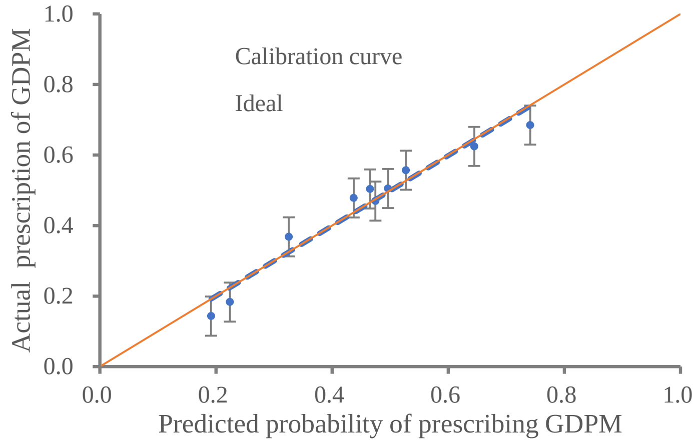

Figure S1 Discrimination and calibration of the prediction model in the total study population. A) AUC of the prediction model. B) Calibration curve for predicted versus observed probability of GDPM prescription. AUC, area under the receiver-operating-characteristics curve; CI, confidence interval; GDPM, guideline-directed secondary prevention medication.

A)

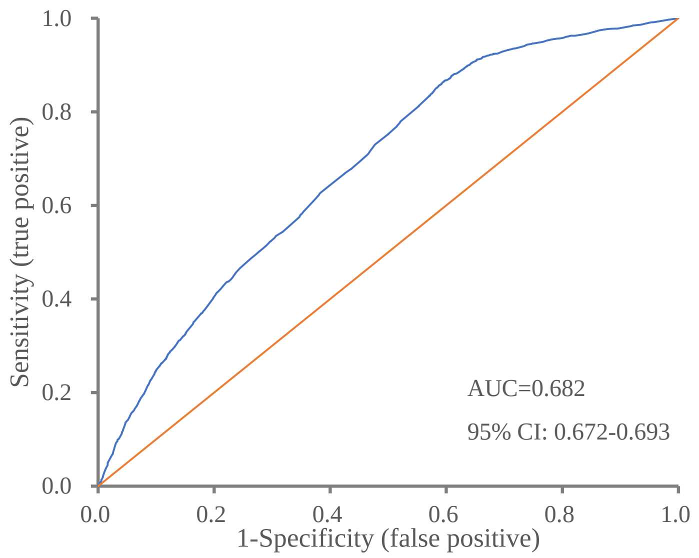

B)

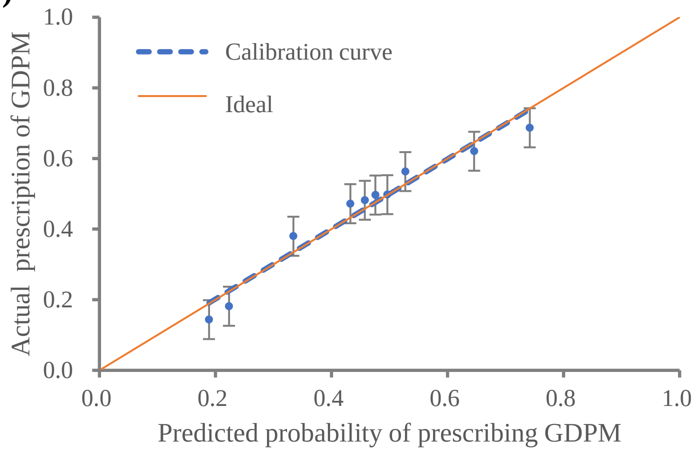

Figure S2 Discrimination and calibration of the prediction model in the cohort of complete cases. A) AUC of the prediction model. B) Calibration curve for predicted versus observed probability of GDPM prescription. AUC, area under the receiver-operating-characteristics curve; CI, confidence interval; GDPM, guideline-directed secondary prevention medication.

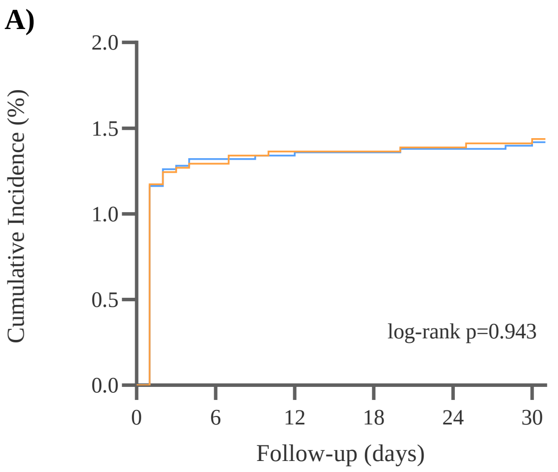

Number at risk

GDPM	4178	4124	4121	4121	4120	4118
non-GDPM	5076	5009	5007	5007	5006	5004

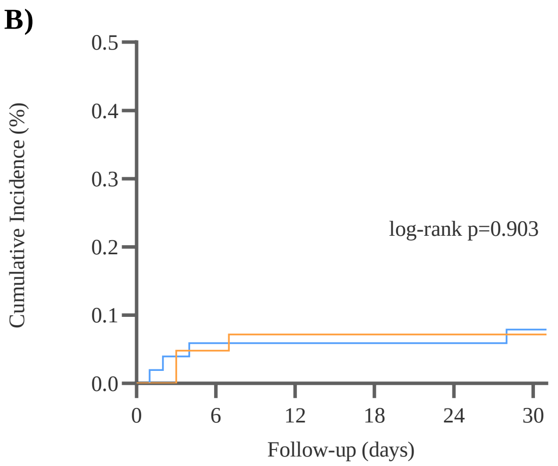

Number at risk

GDPM	4178	4176	4175	4175	4175	4175
non-GDPM	5076	5073	5073	5073	5073	5072

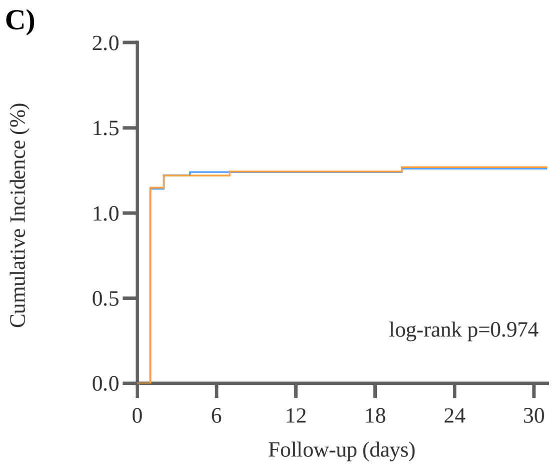

Number at risk

GDPM	4178	4127	4126	4126	4125	4125
non-GDPM	5076	5013	5013	5013	5012	5012

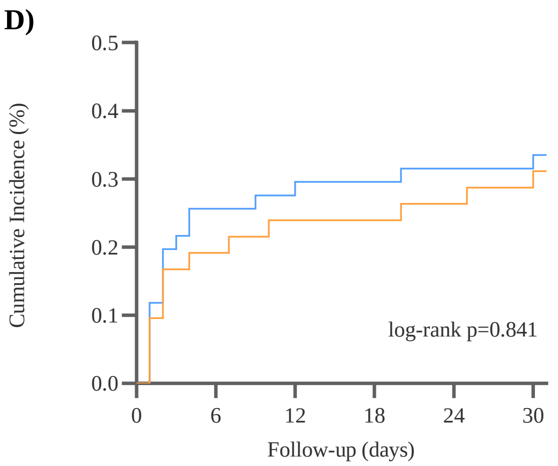

Number at risk

GDPM	4178	4170	4168	4168	4167	4165
non-GDPM	5076	5063	5061	5061	5060	5059

Figure S3 Cumulative incidence curves for 30-day clinical outcomes by prescription pattern for complete cases (A-D) (Orange for GDPM, blue for non-GDPM). Cumulative incidence curves for A) MACE, B) cardiac death, C) MI, D) unplanned revascularization. GDPM, guideline-directed secondary prevention medication; MACE, major adverse cardiovascular event; MI, myocardial infarction.

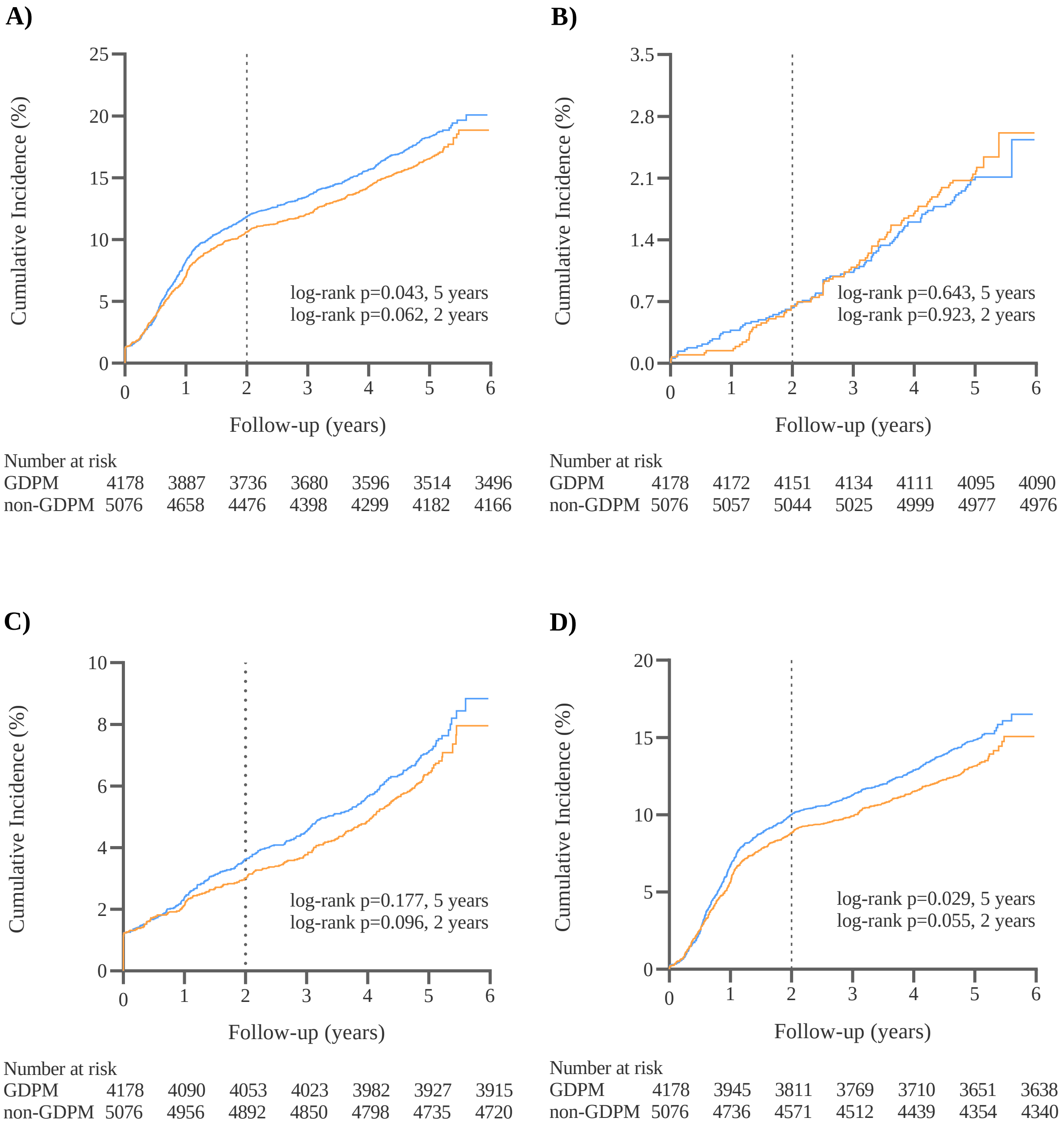

Figure S4 Cumulative incidence curves for 5-year clinical outcomes by prescription pattern for complete cases (A-D) (Orange for GDPM, blue for non-GDPM). Cumulative incidence curves for A) MACE, B) cardiac death, C) MI, D) unplanned revascularization. GDPM, guideline-directed secondary prevention medication; MACE, major adverse cardiovascular event; MI, myocardial infarction.
